# Mapping the knowledge network of extracellular vesicles and angiogenesis in tumor-immune communication: a scientometric analysis

**DOI:** 10.3389/fonc.2025.1568365

**Published:** 2025-07-01

**Authors:** Kai Wang, Dufu Lu

**Affiliations:** Department of Anesthesiology, Honghui Hospital, Xi’an Jiaotong University, Xi’an, Shaanxi, China

**Keywords:** extracellular vesicle, angiogenesis, immunotherapy, cell communication, tumor microenvironment

## Abstract

**Objective:**

This study aims to explore the cooperative networks among authors, nations, organizations, and publications specializing in extracellular vesicle-angiogenesis, to evaluate the foundational knowledge, and to pinpoint future research directions and new topics.

**Approach:**

We extracted all relevant articles from the Web of Science Core Collection (WoSCC) and reviews related to extracellular vesicle-angiogenesis published between January 1999 and December 2023, which met our inclusion criteria. We conducted author and keyword composition analyses using VOSviewer 1.6.18. Furthermore, we utilized Citespace 5.8 to analyze journals, institutions, countries, and keyword timezone maps to elucidate the knowledge network within this field.

**Results:**

The number of academic papers on extracellular vesicle-angiogenesis has steadily increased over the past 25 years. Researchers have closely collaborated globally, with 6,741 papers published in 4,393 academic journals across 111 countries or regions. Shanghai Jiao Tong University has the highest publication volume. Immunology and cancer are the primary focus areas, with *Frontiers in Immunology* and *PLoS One* being significant journals in this field. Cluster analysis results indicate that the relationship between extracellular vesicle-angiogenesis and tumors, inflammation, and regenerative medicine are the three main subfields in this domain. Current research hotspots include mesenchymal stem cells, exosomes, angiogenesis, circulating microRNAs, angiogenesis, and tumor microenvironment.

**Conclusion:**

This bibliometric study utilized visualization techniques to develop a knowledge map of extracellular vesicle-angiogenesis, revealing the significance of extracellular vesicles in tumor evasion by regulating intercellular communication between tumor cells and immune cells, and discussed the potential of exosomal biomarkers in precision medicine.

## Graphical Abstract

Overview of the technical roadmap.

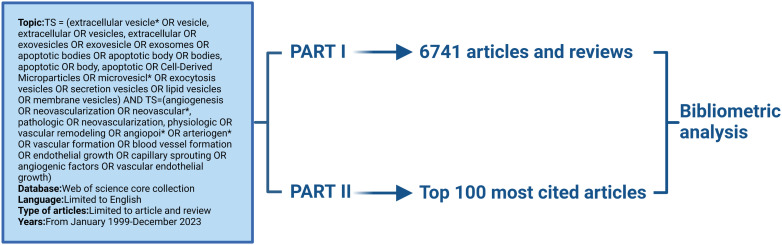

## 1 Introduction

Extracellular vesicles (EVs) encompass a diverse array of vesicles with heterogeneous membrane structures, primarily including exosomes, microvesicles, and apoptotic bodies, originating either from the endosomal system or directly from the plasma membrane (1, 2). Recently, exosomes acting as intermediaries in interactions between the tumor microenvironment (TME) and the immune system have garnered increasing attention in *Cancer Research (* 3–5 *).* These small yet powerful extracellular vesicles act as bridges connecting tumor cells with the immune system, carrying specific proteins, microRNAs, long non-coding RNAs, and other molecules, directly participating in regulating the behavior of immune cells and affecting tumor growth, dissemination, and the capacity for immune evasion (2, 6–8).

Tumor cell-derived exosomes can communicate directly or indirectly with specific immune cells, including effector T cells (Teff), regulatory T cells (Treg), and natural killer (NK) cells, and macrophages, primarily through the interaction of specific molecules on the exosome surface interacting with receptors or ligands on immune cells (9–11). For instance, the interaction between PD-L1 present on the surface of exosomes and PD-1 on T cells can effectively inhibit T cell activity, thereby aiding tumor cells in evading immune system attacks (12, 13). Moreover, specific miRNAs transferred by tumor-derived exosomes, such as miR-21 and miR-155, can regulate the polarization of macrophages toward the M2 type, a subtype with immunosuppressive functions that facilitates tumor growth and metastasis (14–16). Additionally, studies have found that exosomes can affect the maturation and function of dendritic cells (DCs) (17); tumor cell-released exosomes, by carrying tumor antigens and acting on Toll-like receptors (TLRs) on DCs, promote the maturation of DCs and activate specific T cell responses, offering potential for utilizing exosomes as carriers for tumor vaccines (18, 19). NK cells, as crucial immune cells in combating tumors, can have their activity directly influenced by tumor-derived exosomes through the modulation of ligand expression on the NKG2D receptor (20, 21).

Furthermore, extracellular vesicles also promote tumor blood supply and growth by affecting angiogenesis-related targets and signaling pathways. They carry factors like VEGF and FGF that can directly act on vascular endothelial cells to stimulate the formation of new blood vessels (22, 23). These vessels not only provide essential nutrients for the tumor but also create pathways for tumor cell invasion and distant metastasis (24, 25).

These complex interactions reveal the diversified functions of extracellular vesicles in regulating the tumor microenvironment, especially their role in tumor immune evasion mechanisms, providing fresh perspectives for the creation of innovative cancer treatment strategies (26, 27). By deeply exploring the interactions between extracellular vesicles and immune cells, not only can the mechanisms of tumor immune evasion be revealed, but also new prognostic biomarkers could be identified, offering powerful tools for early diagnosis and therapeutic monitoring of tumors (28).

Scientometric serves as a crucial technique for building knowledge networks, revealing hidden logical links among journal articles, such as author collaborations, co-occurrence frequencies of keywords, and the unification of national institutions (29, 30).

Scientometric serves to visualize information, similar to a traditional meta-analysis that compiles unachievable tasks and guides readers in discovering trends and hotspots in a field, thus becoming increasingly important (31, 32). We aim to provide an objective description of the knowledge framework, development patterns, and future projections of the field of extracellular vesicle-angiogenesis using VOSviewer and CiteSpace by (1) quantifying and identifying general information like yearly publications, journals, co-cited journals, regions, authors, and co-cited authors to assess individual impact and collaboration; (2) analyzing the knowledge base of extracellular vesicle-angiogenesis through the examining co-cited references to pinpoint and interpret the most frequently cited papers; (3) more importantly, employing burst analyzing keywords and co-cited references to reveal the development of knowledge frameworks and focal points; (4) and further determining the research focal points, evolution, and potential developmental trajectories of extracellular vesicle-angiogenesis by analyzing the top 100 articles from journals, co-cited journals, countries, and keywords in conjunction with the analysis in part three.

## 2 Materials and methods

### 2.1 Research design

This study analyzed the literature in the field of extracellular vesicle-angiogenesis using Scientometric. The entire section is divided into two parts. The first part is an extensive analysis of papers in the area of extracellular vesicle-angiogenesis. The second part is a study among the top 100 cited articles in extracellular vesicle-angiogenesis (**Graphical Abstract**).

### 2.2 Data collection

The most commonly used database for bibliometric research is the Web of Science Core Collection (33–36). We selected it because it provides us with the comprehensive information we need and is recognized as the leading database. We retrieved relevant paper information from this database on January 2, 2024. Detailed search strategies are provided in the **Supplementary Materials**. Part I: Obtain papers from the setting up of the Web of Science Core Collection to 2023/12/31. Part II: Obtain The 100 most frequently cited papers. We included articles and reviews written in English in our search results. The “Full Record and Cited References” information for each included article was downloaded in plain text format. The file was then saved with the name “download*.txt” to be recognized and further analyzed by CiteSpace.

### 2.3 Data analysis and visualization

Bibliometric analysis relies on specialized software such as VOSviewer developed by Leiden University, CiteSpace developed by Drexel University, SCI2 developed by Indiana University, NetDraw developed by Analytic Technologies, and HistCite developed by Thomson Reuters. Among these five software tools, CiteSpace is commonly used for knowledge mapping analysis, while VOSviewer is suitable for constructing and visualizing networks of large bibliographic databases (37, 38). Therefore, we chose to use both VOSviewer and CiteSpace for analysis (39). We used VOSviewer 1.6.18 to depict the publication volume and co-citation relationships of journals and authors. Based on the included data, we created knowledge maps and generated co-occurrence and cluster graphs from textual data.

Initially, we cleanse the data; for instance, we unify “exosome” to “exosomes” in keyword analysis. Secondly, we applied fraction counting as the method of tallying and limited the maximum author account to 25. The distinction between whole counting and fraction counting lies in the strength of links. The fraction counting method calculates link strength based on the weight of the paper. For example, given that three authors co-authored an article, each link’s strength counts as 1/3 in the fractional count but 1 in the total count. The fractional count method behaves reasonably, and after a more extensive comparison of the remaining data, our data act better in rational and clarity in the fractional count method.

CiteSpace, developed by Drexel University, is commonly used for visualizing data that explores partnerships, key terms, internal organization, emerging trends, and advancements. Consequently, we used CiteSpace 5.8 to perform analysis and visualization of national and regional co-occurrence patterns, journal biplots, trends in high-centrality keywords, co-cited references, and bursts in references (40). Before this, we also cleaned the data. For instance, publications from “.USA” were classified under “USA”. In a similar manner, we unified “exosome” to “exosomes” in the keyword analysis. The parameters of CiteSpace were set as follows: time span set from 1999 to 2023, one year per slice, pruning applied using minimum spanning tree and pruned slice network, selection criteria set to top N=50, and all other settings kept at their default values. We utilize Excel 365 for database management and publication analysis, and additionally, we obtained the IF and JCR ratings for journals. We employ Microsoft Office Excel 365 for database management and publication analysis.

## 3 Results

### 3.1 Publication trends and annual citation rates

**Figure 1:**
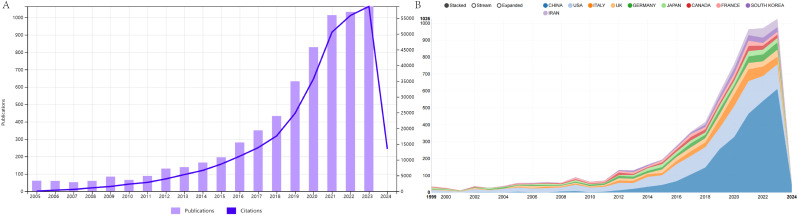
**(A)** Temporal distribution map of publications and citations of Review and Article from 1999 to 2023. **(B)** The country’s annual trend publications related to extracellular vesicles- angiogenesis from 1999 to 2023.

Following our collection strategy, we gathered a total of 6951 papers. After limiting the publication types to articles and reviews and restricting the language to English, a total of 6,741 publications were included in the study. As displayed in **Figure 1A**, the number of references related to extracellular vesicle-angiogenesis has steadily increased from year to year. Furthermore, we examined the top10 countries of publication. In 2017 and before (1999-2017), the United States emerged as the leading publisher of studies, but in 2018-present, China has demonstrated significant interest in extracellular vesicle-angiogenesis, surpassing the United States to become the country with the highest number of publications in this field (**Figure 1B**).

### 3.2 Journals and co-cited journals

To identify the most prolific and influential journals in this field, we used VOSviewer to analyze the co-cited references. The data showed 6,741 papers published in 1,283 academic journals. References published by *Frontiers in Immunology* (IF 2023 = 7.3) (N=68, 1.16%) ranked in the top 10, indicating the influence of *Frontiers in Immunology* in the field of extracellular vesicle-angiogenesis and their essential contribution to the advancement of the area. Five of the top 10 journals belong to Switzerland and the remainder to the US and UK. The top 10 journals make up approximately 17.2% of the total published articles,7 of which are in the Q1 region of the JCR, and 3 have an IF above 5, as indicated in **Supplementary Table 1**.

Among all the journals, 10 had more than 2,000 co-citations, with *PLoS One* ranking the highest (N=3493) (**Supplementary Table 2**). This is followed by the *Proceedings of the National Academy of Sciences of the United States of America*, *Journal of Biological Chemistry*, *Nature*, and other journals. Among the top 10 journals with the highest co-citation counts, 6 have an impact factor exceeding 10 and 9 are classified in the JCR Q1 quartile. The highest impact factor among them is 50.5. It is noteworthy that most of these high-impact journals are based in the United States.

**Figure 2:**
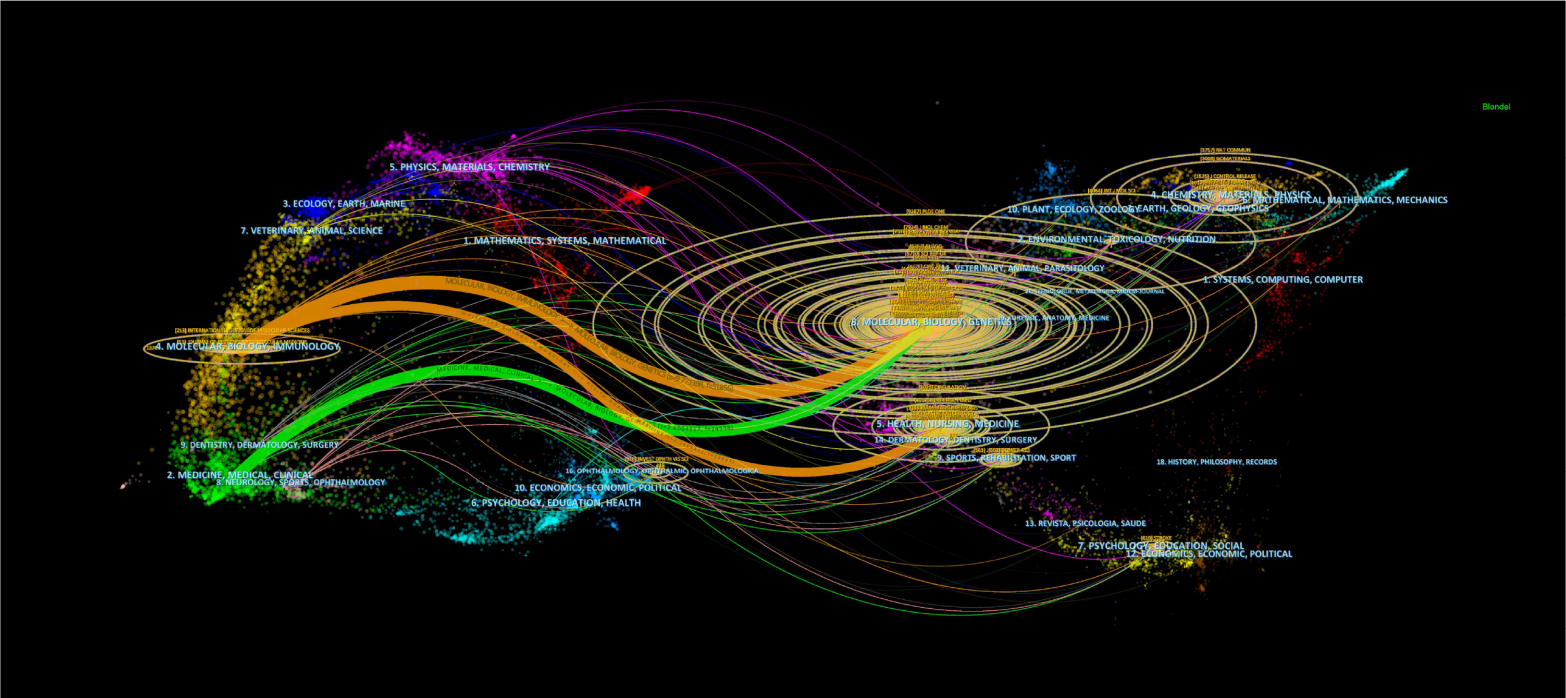
Biplot overlay of journals related to extracellular vesicle-angiogenesis studies. The citing journals are on the left, the cited journals are on the right, labels represent the disciplines covered by the journals, and colored paths represent citation relationship between countries/regions and organizations.

**Figure 3:**
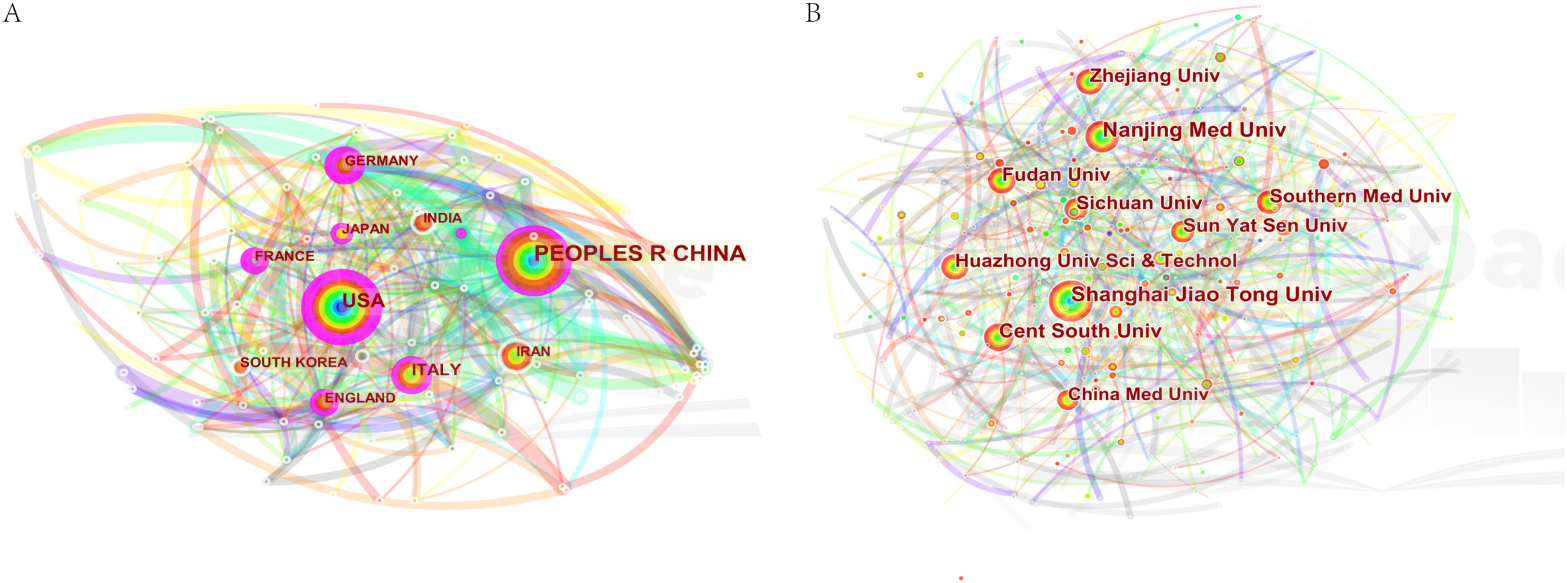
Co-occurrence diagram of **(A)** countries/regions and **(B)** institutions in extracellular vesicle-angiogenesis studies. Notes. The size of the nodes reflects the frequency of co-occurrence, and the links indicate the co-occurrence relationships. The colors of nodes and lines represent different years, ranging from purple to red, from 1999 to 2023; purple rounded nodes imply high betweenness centrality (>0.20).

The double chart of the journal is overlaid to show the distribution of topics. From left to right, lines of different colors represent three specific citation paths. The two orange citation paths illustrate that articles from Molecular/Biology/Genetics journals, specifically those focused on molecular mechanisms and genetic research, and Health/Nursing/Medical journals, including studies on healthcare practices and patient care, are frequently referenced in Molecular/Biology/Immunology journals. The green path demonstrates that research from Molecular/Biology/Genetics journals, particularly studies related to genetic engineering and molecular biology, is often cited in Medical/Clinical journal studies (**Figure 2**). There were 6,741 papers from 670 institutions across 111 countries/regions, with **Supplementary Table 3** showing the top 3 countries/regions as China (n=2137, 35.11%) USA (n=939, 15.43%), and Italy (n=312, 5.13%). As shown in **Figure 3A**, certain nodes, like the United States, France, and Germany, are highlighted by purple circles representing high centrality (≥0.20), and nodes with centrality above 0.1 are often considered critical pivotal points that could facilitate groundbreaking discoveries and serve as bridges to awaken. In addition, the United States (1999), Australia (1999), and the United Kingdom (1999) were among the first countries to perform extracellular vesicle-angiogenesis related studies. We utilized the settings “minimal spanning tree” and “pruned slice network” to simplify and clarify the network structure. The unsliced country co-occurrence graph includes 81 nodes with a density of 0.1164 and extends 377 links, indicating the active collaboration present among different countries or regions. As shown in **Figure 3B**; **Supplementary Table 3**, all of the top ten institutions are from China (10/10). The top five institutions are Shanghai Jiao Tong University(2.93%), Nanjing Medical University(2.50%), Central South University(2.29%), Huazhong University of Science and Technology(1.85%) and Fudan University(1.83%).

### 3.3 Authors and co-cited authors

**Figure 4:**
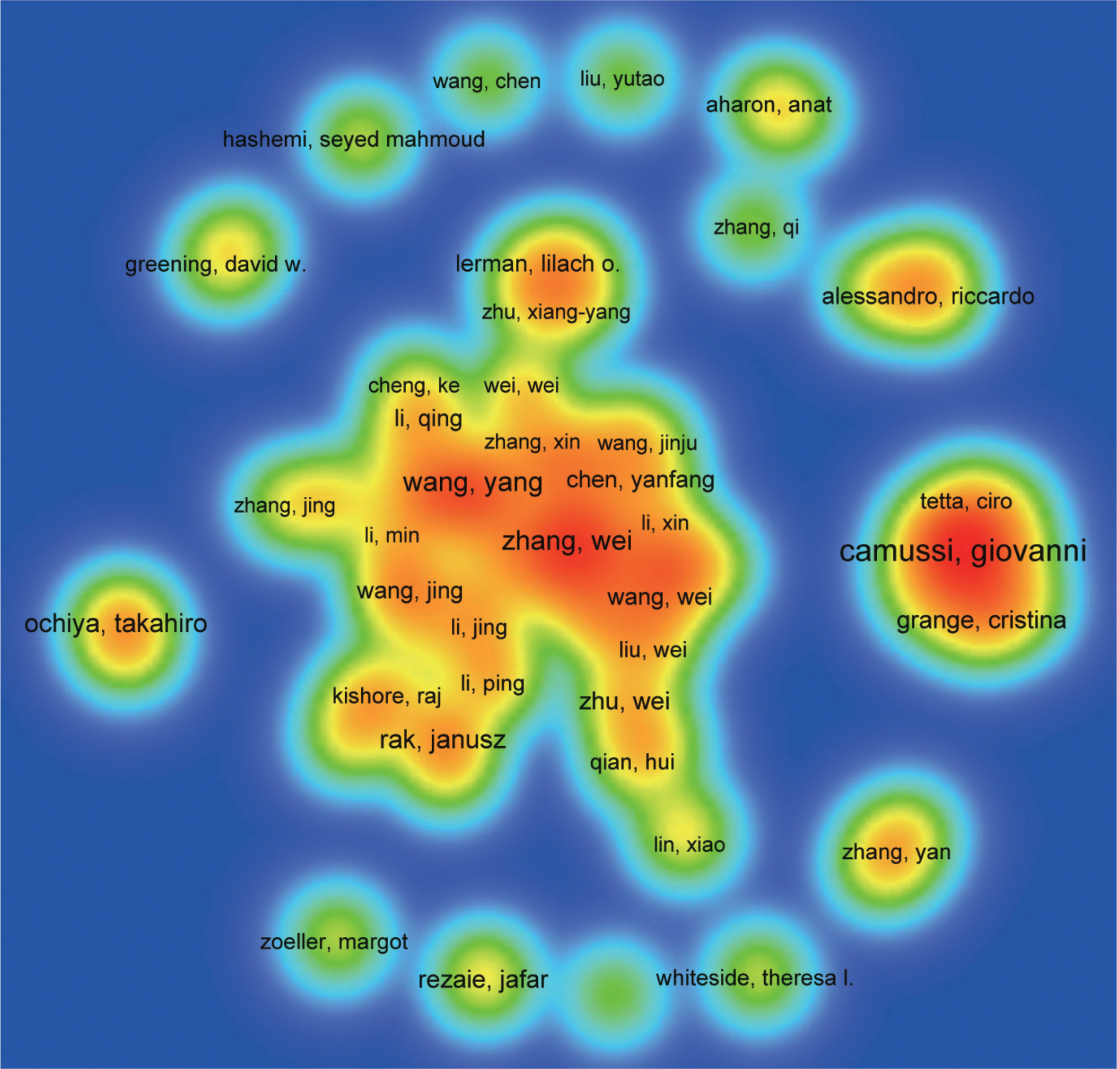
The density map of authors in extracellular vesicles- angiogenesis research. The size of the word, the size of the round, and the opacity of yellow are positively related to the publication frequency.

A total of 31,280 individuals took part in extracellular vesicle-angiogenesis studies. Eight researchers published more than 20 articles. As shown in **Supplementary Table 4**, Giovanni published the highest number of papers (n=48), followed by Wang, Yang (n=28), Zhang Wei (n=28). We developed a knowledge graph according to authors with more than five publications (**Figure 4**), which shows that the high frequency of authors is evident. As depicted in **Figure 4**, Camussi, Giovanni; Tetta, Ciro and Grange, Cristina; three authors are closely linked and form an author group with the darker color, indicating that they contribute a lot to the field of extracellular vesicle-angiogenesis. Wang, yang; Zhang, Wei and Wang, Wei constitute the second-ranked group of authors.

**Figure 5:**
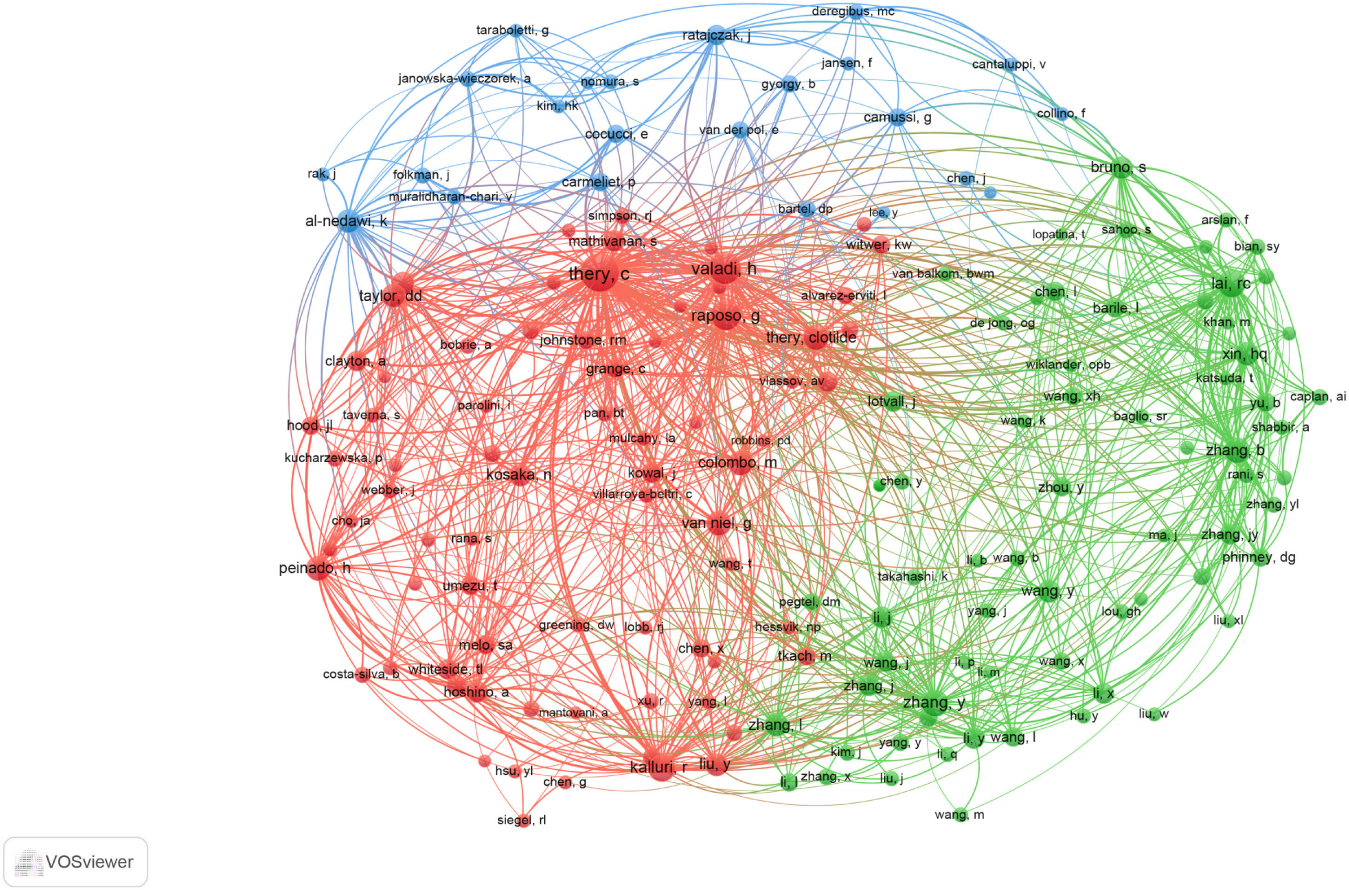
The co-occurrence map of co-cited authors in extracellular vesicle-angiogenesis research. The link indicates the co-occurrence relationship between authors, and the same node color represents the same cluster.

Co-cited authors are those who have been frequently cited together across various publications. Among them, 10 had more than 500 co-citations, as shown in **Supplementary Table 4**. Thery C ranked first (n=1349), followed by Baladi H (n=909); Raposo G (n=796); Lai, RC (n=668); Zhang Y (n=662). Authors with a minimum of 50 (T ≥ 50) co-citations were selected to draw the co-citation author network (**Figure 5**), with the same color indicating the same group. The co-cited authors were categorized into three main clusters. Authors within the same cluster exhibited close collaboration, such as Thery C, Valadi H and Raposo G in the field of extracellular vesicle-angiogenesis. Also, we noticed significant cooperation among clusters such as Thery C, Valadi H and Zhang Y.

### 3.4 Keywords co-occurrence, clustering, and evolution

**Figure 6:**
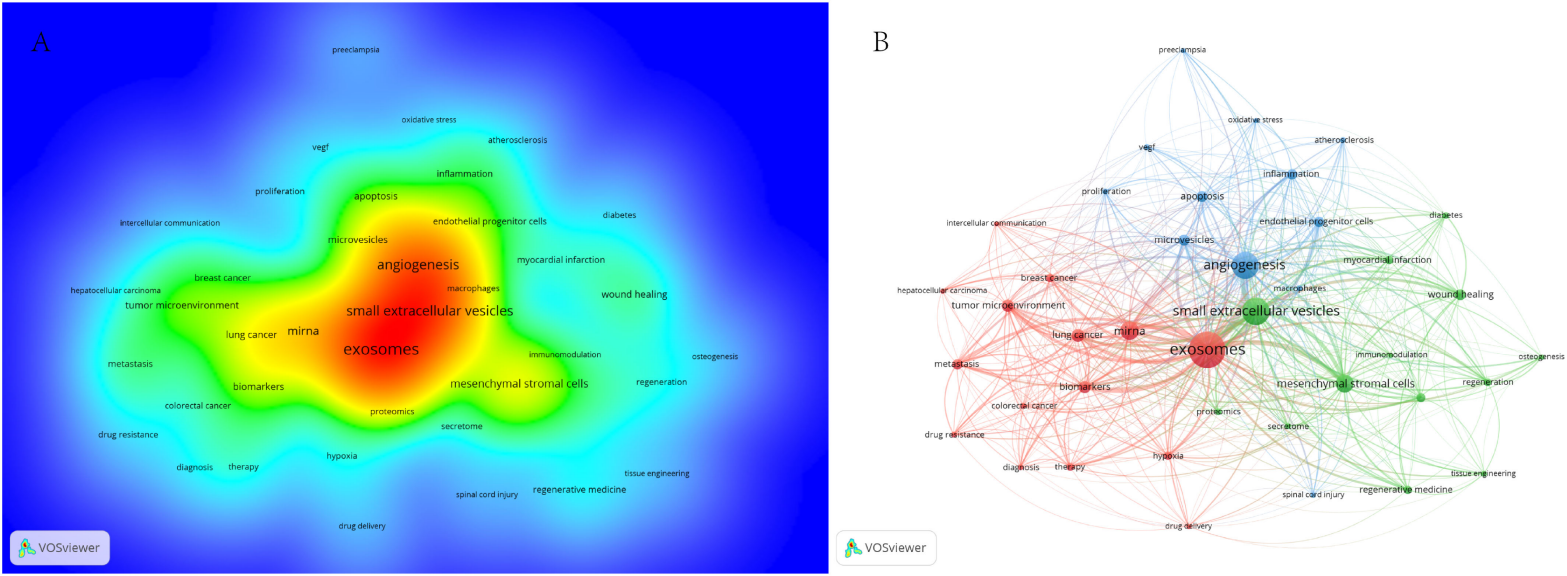
**(A)** Term density maps in extracellular vesicle-angiogenesis studies. The size of the term, the size of the circle, and the opacity of the red color are positively correlated with the frequency of co-occurrence. **(B)** Keyword co-occurrence networks and clusters in extracellular vesicle-angiogenesis studies. Remarks. Node and word sizes reflect co-occurrence frequency, links indicate co-occurrence relationships, and nodes of the same color represent the same cluster.

To identify terms with high-frequency co-occurrence and uncover hotspots in specific research areas, we conducted keyword co-occurrence and cluster analysis using VOSviewer (**Supplementary Table 5**; **Figure 6**), generating a keyword density visualization (**Figure 6A**) (41). From this analysis, we extracted 7,190 keywords, with 20 keywords appearing more than 200 times and 11 keywords occurring more than 100 times. Cluster analysis reveals the knowledge structure within the study area. Based on the co-occurrence link strength of lexical items, we divided the network into three clusters. There is a high degree of homogeneity among the lexical items in a cluster. As shown in **Figure 6B**, cluster 1 (red) is the largest cluster and includes 15 items. Exosomes, miRNAs, biomarkers, tumor microenvironment, cancer, chemoresistance, therapy, etc. Cluster 1 focuses on the role of extracellular vesicles in tumors. Cluster 2 (green) addresses 13 elements related to extracellular vesicles and regenerative medicine. Cluster 3 (blue) focuses on the role of extracellular vesicles in inflammation.

**Figure 7:**
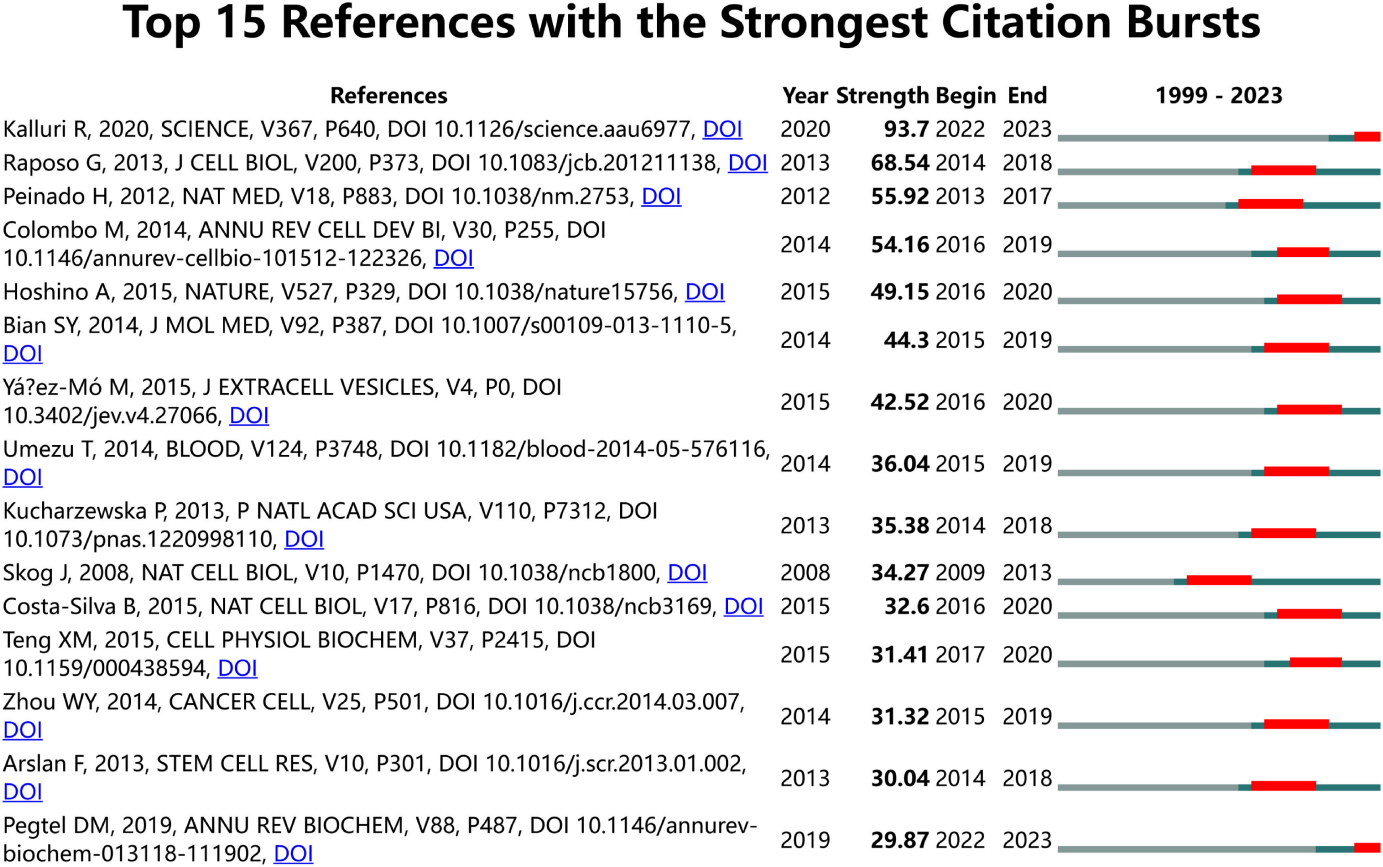
Display the two keywords with the highest centrality each year. Links represent co-occurrence relationships. The color of the lines indicates temporal information. Gray to red represents the years from 1999 to 2023.

To describe the research focus and development trends, we used CiteSpace to select the two most central keywords for each year to create a time zone diagram (**Supplementary Table 7**; **Figure 7**), with the colors of the connections and horizontal axis labels indicating the specific years in which the keywords appeared. In the diagram, we observed changes in high-centrality keywords across different years. High-centrality keywords from the past three years often indicate future hotspots; they are neuroinflammation, tumor microenvironment, and safety, which hold significant positions.

### 3.5 Co-cited references and citation bursts

**Figure 8:**
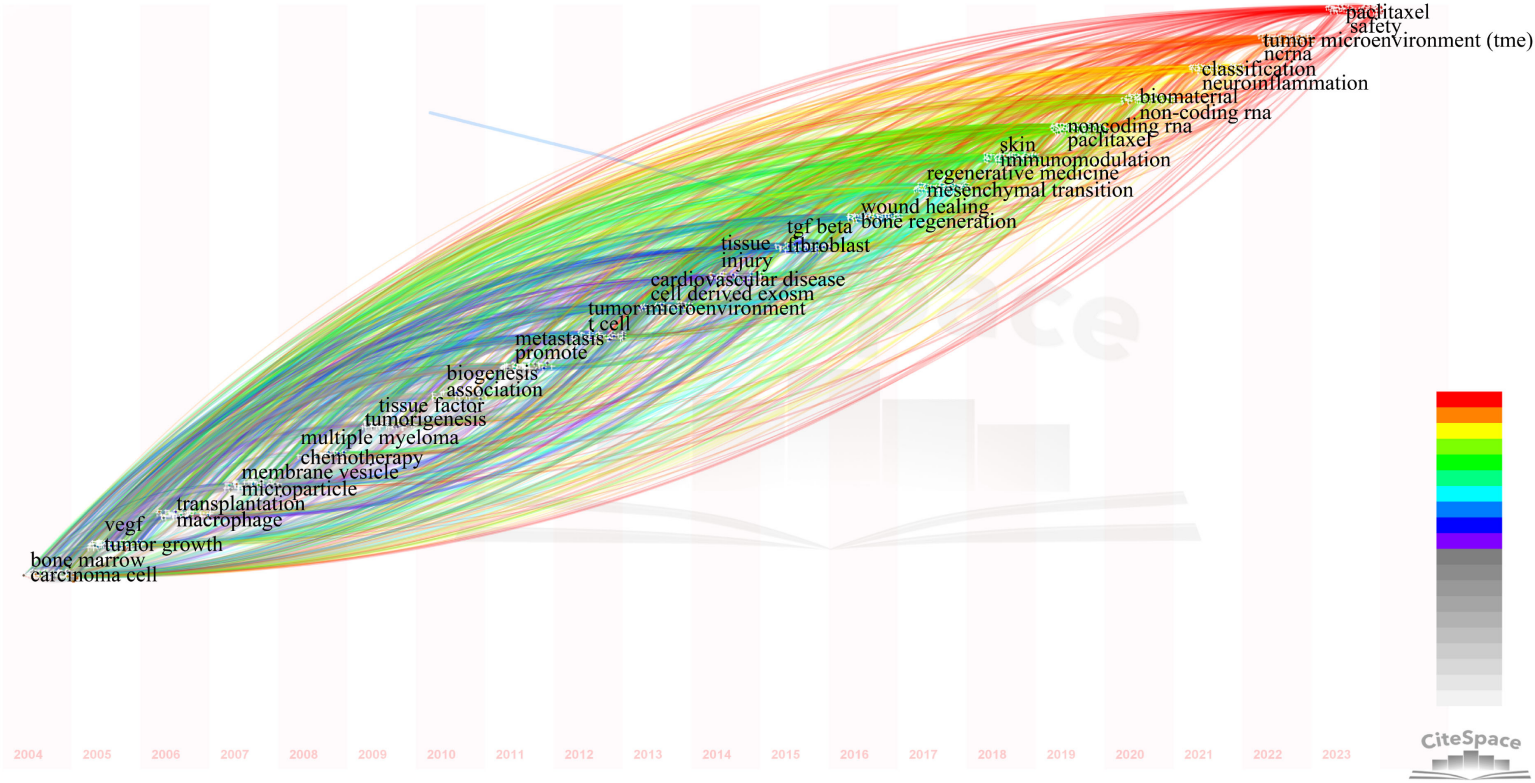
Visual analysis of references bursts. The strength reflects the cited frequency. The red bar indicates citation frequency; the green bars indicate fewer citations.

We utilized CiteSpace for identifying co-cited references (**Supplementary Table 6**). Among the top 10 co-cited references, the lowest number of co-citations was 190, while the highest exceeded 500. This reference was published by Graça Raposo in 2013 in the *Journal of Cell Biology* titled “Extracellular vesicles: Exosomes, microvesicles, and friends” (1). A citation burst is defined as a reference that experiences a sudden significant increase in citations over a specific period (42). Using CiteSpace, We identified citation bursts that persisted for at least one year. We chose the top 15 strongest references for analysis (**Figure 8**). Most of the references of the burst citations were published after 2010. Notably, two references (14%) remained in burst status until 2023.

### 3.6 Analysis of the top 100 most cited documents, journals, countries, and keywords

**Figure 9:**
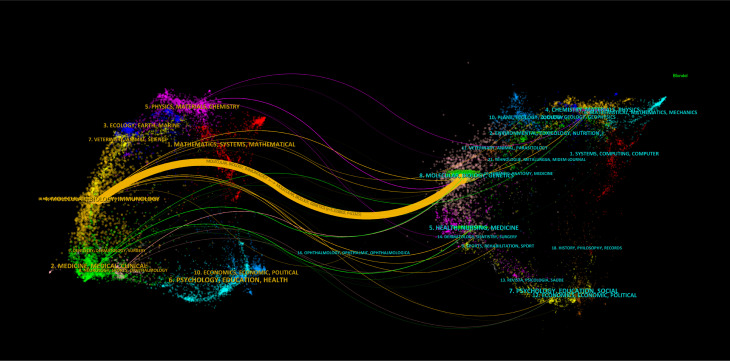
Biplot overlay of the top 100 most cited journals associated with extracellular vesicle-angiogenesis studies. The citing journals are on the left, the cited journals are on the right, and colored paths represent citation relationships.

The TOP100 highly cited literature is the TOP100 highly cited literature related to extracellular vesicle-angiogenesis research. We examined the journals with TOP100 articles and co-cited journals (**Supplementary Tables 8**, **9**; **Figure 9**).

**Figure 10:**
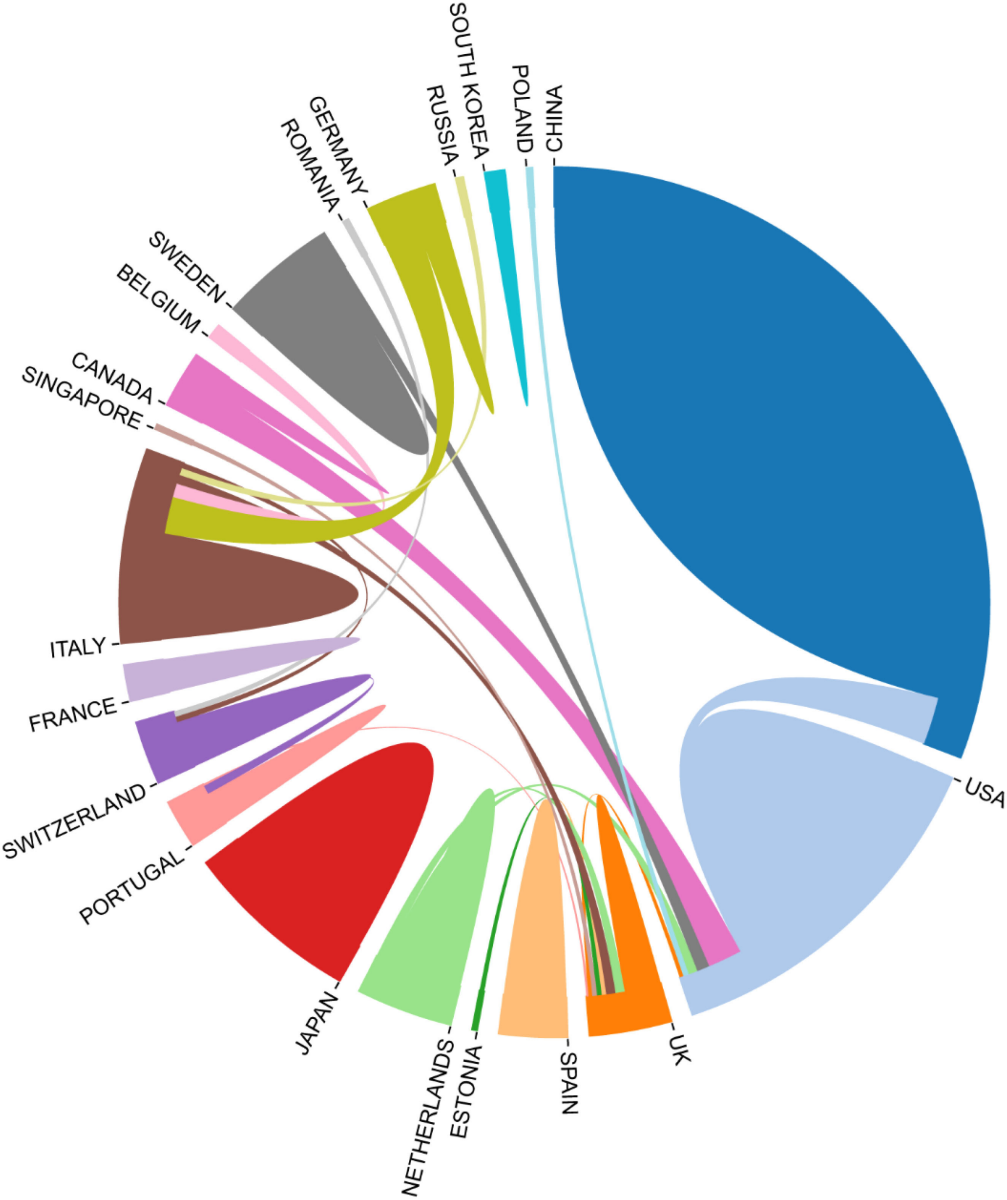
Country-to-country relationship for the top 100 most cited articles.

Fourteen journals each published over three articles, with *Blood* (n=4) and *Frontiers in Physiology* (n=4) tied for the top ranking. Among the co-cited journals, *Blood* (n=221) ranked first. The single orange citation path highlights that research published in molecular/biology/immunology journals is predominantly cited by studies originating from molecular/biology/genetics journals. Additionally, when analyzing the countries of the top100 papers, China has the highest number of papers, with USA following behind (**Figure 10**).

**Figure 11:**
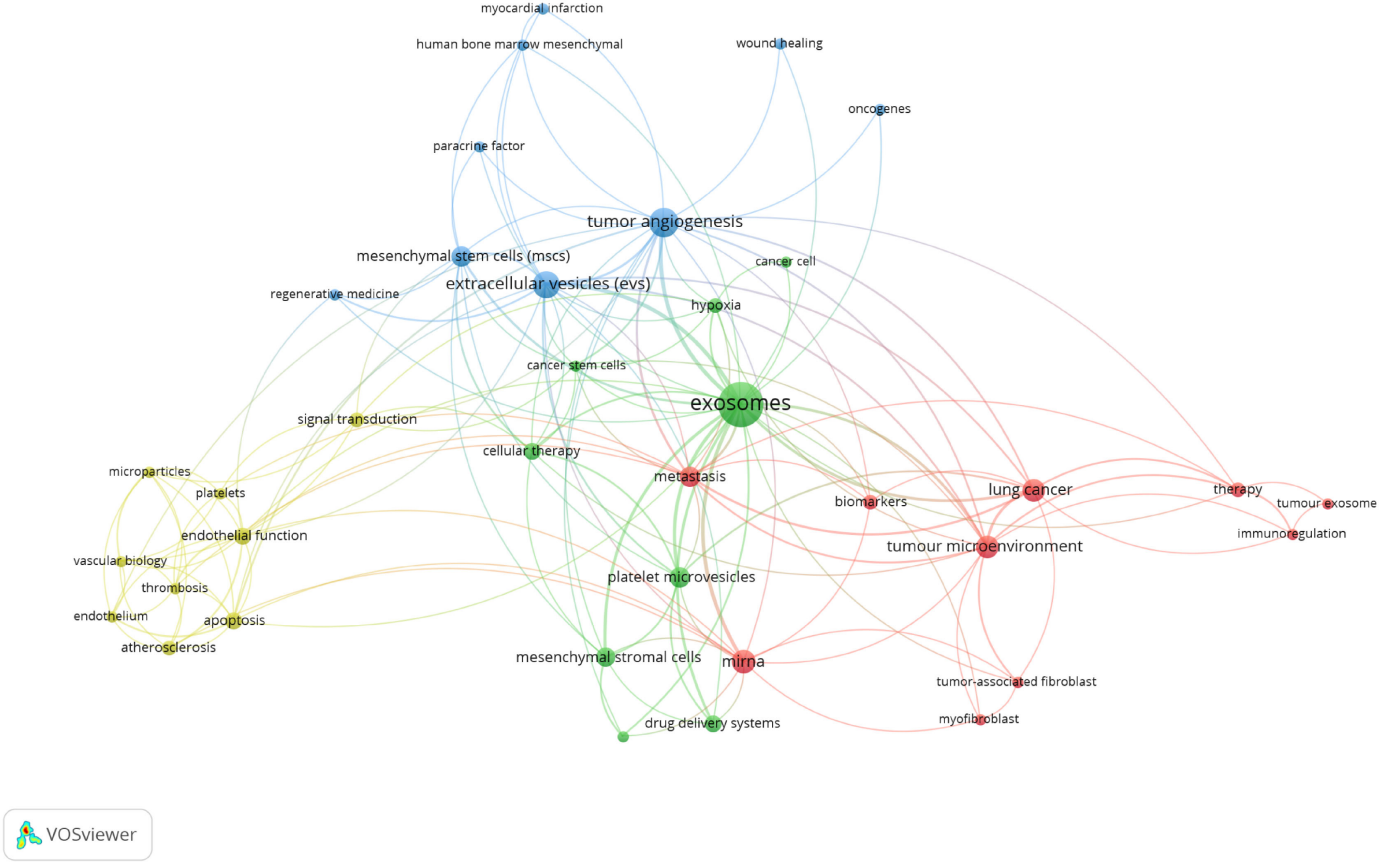
Keyword co-occurrence network and clusters in extracellular vesicle-angiogenesis studies. Remarks. Node and word sizes reflect co-occurrence frequency, links indicate co-occurrence relationships, and nodes of the same color represent the same cluster.

There are regular academic exchanges between China and the US, with the US also maintaining contacts with 13 countries, including the UK, the Netherlands and Canada, and Poland. To further identify research trends and hotspots in this field, we extracted the keywords from the top 100 cited articles. The top 20 keywords (**Supplementary Table 10**) overlap with those of the field of extracellular vesicle-angiogenesis (**Supplementary Table 5**), such as exosomes, angiogenesis, microvesicles, extracellular vesicles, mesenchymal stem cells, and hypoxia. This suggests that the study of extracellular vesicle-angiogenesis mainly focuses on the fields mentioned earlier. The network is divided into 4 clusters according to the strength of the links for the co-occurrence of words (**Figure 11**).

Cluster 1 (red) is the largest, involving tumor immunoregulation, including biomarkers, immunoregulation, lung cancer, metastasis, mirna, etc. Cluster 2 (green) involves tumor therapy, including cancer cell, cancer stem cells, cellular therapy, drug delivery systems, exosomes, etc. Cluster 3 (blue) focuses on the relationship between exosomes and regenerative medicine, including myocardial infarction, oncogenes, paracrine factor, regenerative medicine, tumor angiogenesis, etc. Cluster 4 (yellow) involves exosomes and vascular biology, including microparticles, platelets, signal transduction, thrombosis, vascular biology, etc.

## 4 Discussion

### 4.1 General information

According to the WoSCC database as of 2023/12/31, 4,393 authors from 111 countries/regions 31,280 authors from 670 institutions have published 6741 studies on extracellular vesicle-angiogenesis in 12,803 journals. Changes in the annual publication volume and citation counts are crucial indicators for revealing the development trends in the field. Studies related to extracellular vesicle-angiogenesis were presented in Denzer, K et al. (43) This article discusses the journey of exosomes from internal vesicles within multivesicular bodies to their role as intercellular signaling devices. Subsequently, there has been a general upward trend in articles related to extracellular vesicle-angiogenesis (**Figure 1**). The articles related to extracellular vesicle-angiogenesis can be divided into three phases: the “ Initial Development Phase,” the “ Consistent Growth Phase,” and the “ Accelerated Expansion Phase.” Initial Development Phase” (1999-2010): the concept of extracellular vesicle action in angiogenesis was formally introduced (44), producing no more than five articles annually over these 12 years.” Steady growth” (2011-2018): during this period, extracellular vesicle-angiogenesis received more scientific attention, and the annual production increased steadily.” (45, 46) Rapid growth” (2019-present): during this period, not only has the number of annual articles steadily increased, but publications have also garnered higher citation rates than those before 2019, indicating that extracellular vesicle-angiogenesis research is gaining interest from researchers and overgrowing (47–49). Furthermore, the growth trend looks promising.

The analysis of journals and co-cited journals (**Supplementary Tables 1**, **2**) showed that *Frontiers in Immunology* ranked in the top 10 in terms of production in the field on extracellular vesicle-angiogenesis, implying that *Frontiers in Immunology* has a long-standing interest in the field of free extracellular vesicle-angiogenesis and has a significant presence in the area. *PLoS One*, on the other hand, received the most co-citations. Both journals focus on cell biology, as supported by the biplot analysis (**Figure 2**). The journal biplot overlay depicts the disciplinary distribution of academic journals; (**Figure 2**) shows the three main citation paths from molecular/biology/immunology co-citation journals to molecular/biology/genetics journals, one of which is from molecular/biology/immunology co-citation journals to molecular/biology/genetics journals. In addition, journals ranked in the Q1 region in the JCR partition represent most of the top 10 journals (70%) and co-cited journals (90%), highlighting the interest and role of these journals in research related to extracellular vesicle-angiogenesis.

There are differences in extracellular vesicle-angiogenesis studies between countries/institutions (**Supplementary Table 3**; **Figure 3**). The United States, China, and Italy are the leading 3 producing countries and are considered significant turning points that have the potential to drive groundbreaking discoveries. Additionally, the United States, Germany, and France carried out the initial extracellular vesicle-angiogenesis studies, followed by Italy, Japan, and Switzerland, which were also in the top 10 producing countries. Notably, despite a late start, China has become one of the foremost contributors to productivity in recent years, which could be associated with the economic development of the country and the financial investment in academic research. Additionally, there are active collaborations among various countries/regions, especially in the United States, suggesting that extracellular vesicle-angiogenesis related research has garnered global attention, with the United States being the main center of collaboration.

Next, let us focus on influential researchers in this field and the collaboration among authors. Authors with high co-citations and publication volumes usually hold significant positions in the field and can offer valuable guidance and direction to scholars. Our analysis (**Supplementary Table 4**; **Figures 4**, **5**) shows that Camussi, Giovanni, and Thery, C have made important contributions to this field. The former two have the highest publication volumes, while the latter has the highest number of co-citations. Additionally, the collaboration relationships between authors and co-cited authors reveal the cooperative dynamics within the field. Notable collaborations exist among authors from different institutions, particularly among those holding key positions in the field. For example, 54 researchers from 35 institutions published a weighty treatise entitled “Tumour exosome integrins determine organotypic metastasis.” (50) This indicates that high-impact collaborative groups composed of researchers are widespread in this field.

### 4.2 Knowledge base

Co-cited references are multiple references that are cited together in the same document. They are different from highly cited references in definition and constitute the knowledge base of the research field (51, 52). The top 10 co-cited references in the extracellular vesicle-angiogenesis literature are included in this bibliometric analysis (**Supplementary Table 6**). In 2013, *Journal of Cell Biology* published the most co-cited study co-authored by Graça Raposo et al. (1) A paper that describes the formation of exosomes, their involved functional processes, and mechanisms points out that cells within the tumor microenvironment, similar to tumor cells, have the capability to secrete exosomes. These exosomes play a crucial role in promoting tumor progression by enhancing tumor angiogenesis and the movement of tumor cells. In 2008, Johan Skog et al. published a paper in *Nature Cell Biology* on glioblastoma cells releasing exosomes containing mRNA, miRNA, and angiogenic proteins, which could not only provide diagnostic information for cancer patients to make therapeutic decisions through blood tests but also determined that glioblastoma-derived exosomes contribute to angiogenesis through *in vitro* angiogenesis assays (53).

The third co-cited article was published by Marina Colombo et al. in 2014 in *Annual Review of Cell and Developmental Biology*, also pointing out that tumor exosomes are involved in tumor cell metastatic dissemination and also exhibited pro-angiogenic activity interacting with endothelial cells (54). The fourth most co-cited paper was published by Héctor Peinado et al. in 2012 in *Nature Medicine*. This study revealed that melanoma, after intervention with exosomes, undergoes bone marrow angiogenesis mediated by key molecules c-Kit+ and Tie2+, leading to melanoma metastasis and ultimately resulting in the widespread distribution of the tumor (55). In 2015, María Yáñez-Mó et al. published the fifth co-cited study in the *Journal of Extracellular Vesicles* that showed the elaboration of pro- or anti-angiogenic therapies involving exosomes for organ regeneration or cancer treatment (56). The sixth paper was published by Samir EL Andaloussi et al. in 2013 on extracellular vesicles. This study specifically highlights that exosomes play a crucial role in the repair of damaged tissues not only due to their regulatory functions in angiogenesis, apoptosis modulation, and stimulation of cell differentiation and proliferation, but also because they can influence stem cell plasticity (2). In 2014, the *Journal of Molecular Medicine* published a seventh co-cited experimental study by Suyan Bian et al. This study shows that extracellular vesicles from human bone marrow mesenchymal stem cells can enhance angiogenesis and improve heart function after myocardial infarction in rats (57). The eighth article by Suresh Mathivanan et al. in 2010 summarized the role of exosomes in mediating intercellular communication and highlighted the critical importance of tumor progression, angiogenesis, and aggressiveness (58). The ninth co-cited paper of 2011 was published in *Cancer Research* by Cristina Grange et al. This research demonstrates that microvesicles from renal cancer stem cells promote angiogenesis and the establishment of a premetastatic niche in the lungs (59). The 10th co-cited paper of 2007 in *Blood* by Maria Chiara Deregibus et al. The article describes how microvesicles from endothelial progenitor cells induce angiogenesis in endothelial cells through the transfer of mRNA (60).

Overall, the top 10 co-cited references were reviews (5 reviews published in 2010, 2013, 2014, and 2015) and articles (5 treatises published in 2007, 2008, 2011, 2012, and 2014), which are the basis of extracellular vesicle-angiogenesis studies.

### 4.3 Evolution of popular topics, knowledge structure, and emerging topics

In Scientometric, keyword clustering and time zone diagrams effectively indicate research hotspots and their changes and describe the internal knowledge structure of the discipline, revealing the research frontiers (61–63). Cluster analysis revealed three main clusters in extracellular vesicles-angiogenesis research (**Figure 6**), representing the three main aspects: elements of extracellular vesicles, the basis of angiogenesis, and translational therapeutic applications. Extracellular vesicles are membrane vesicles of varying sizes secreted by cells. They are used in clinics as indicators of disease diagnosis, prognosis recovery, and beneficial treatment methods due to their content of various physiopathological signaling molecules and stability in the circulatory system (8, 64). With decades of significant progress in researching the mechanisms of angiogenesis, it has been shown to be crucial in many diseases where new vessels can serve as scaffolds to guide axonal regeneration, supply blood and oxygen to injured areas, and provide the necessary nutrients for repair (48, 65). However, new vessels provide oxygen and nutrients to tumor growth and conditions for tumor metastasis in the tumor development process (47, 66). Therefore, understanding and researching the biological functions of EVs hold promise for diagnosing and treating angiogenic diseases, which is one of the key areas for future research in extracellular vesicles-angiogenesis.

In our study, we employed CiteSpace to extract the two keywords with the highest centrality each year, aiming to illustrate the evolution of high-centrality keywords over the past twenty years (**Supplementary Table 7**; **Figure 7**). To predict future research hotspots, this study utilized Citespace to extract the top two central keywords annually from the past twenty years in this field. By combining Citespace and VOSviewer analysis results, the evolution of high-centrality keywords over nearly 20 years can be divided into five stages.

Stage 1 (1999-2004): This is the initial stage of research in extracellular vesicle-angiogenesis. Researchers began exploring how cancer interacts with immune cells and the vascular system, focusing on fundamental elements related to cancer and immune response, such as “apoptosis,” “expression,” “cancer,” “endothelial cells,” and “bone marrow.” Immune cells and the vascular system were central to the field during this period.

Stage 2 (2005-2009): Research delved into the mechanisms behind tumor growth and immune response, particularly the role of extracellular vesicles in the tumor microenvironment and between immune cells. Key terms included “VEGF” (vascular endothelial growth factor), “tumor growth,” “macrophages,” “multiple myeloma,” and “chemotherapy.”

Stage 3 (2010-2014): During this period, terms such as “biogenesis,” “tissue,” “injury,” “tissue factor,” and “tumorigenesis” became increasingly important. This reflects a broader understanding of the microenvironment beyond just tumors, with a growing interest in the role of extracellular vesicles in cardiovascular diseases, indicating an expansion in the scope of extracellular vesicles research.

Stage 4 (2015-2020): Key terms during this stage included “TGF beta,” “fibroblasts,” “non-coding RNA,” “biomaterials,” and “immunoregulation.” These keywords highlight the precise roles of extracellular vesicles in immune contexts, particularly in specific pathways and cellular processes, and the role of non-coding RNAs in immune regulation and tissue regeneration.

Stage 5 (2021-2023): In 2021, terms like “classification” and “neuroinflammation” indicate that researchers began classifying specific inflammatory processes in the nervous system mediated by extracellular vesicles, suggesting an exploration into the precise role of extracellular vesicles in neurodegenerative diseases and a shift toward precision medicine. In 2022, the reemergence of terms such as “tumor microenvironment (TME)” and “ncRNA” underscores the renewed focus on the function of extracellular vesicles in the tumor microenvironment, emphasizing their importance in intercellular communication within tumors. In 2023, the appearance of “paclitaxel” and “safety” suggests that extracellular vesicles -related research is beginning to focus on clinical translation, particularly the safety and efficacy of therapeutic delivery systems involving extracellular vesicles.

Co-cited references are those that multiple references are frequently cited together in a single document, typically recognized as highly relevant sources in the field. Burst-cited references are those that experience a sudden surge in citations within a specific timeframe, often indicating emerging research hotspots. The knowledge base is comprised of a collection of pivotal literature in the field, reflecting critical achievements and the core knowledge system (67).By intersecting the top 10 co-cited references and the top 15 burst-cited references, we identified six key papers forming the knowledge base in this field.

To more clearly illustrate the relationships among various aspects of knowledge in this domain, we categorized these six articles into three clusters. In 2013, Graça Raposo et al. published a paper on *the Journal of Cell Biology*. This study demonstrated the feasibility of extracellular vesicles (including exosomes and microvesicles) carrying proteins, lipids, and RNA to influence the functions and behaviors of immune cells, establishing the function of exosomes in intercellular communication (1). In 2014, Marina Colombo et al. published an article in the *Annual Review of Cell and Developmental Biology*. This study defined exosomes and other secretory extracellular vesicles, their secretion, and function. It highlighted their role in transmitting proteins, lipids, and RNA capable of modulating immune responses under normal and pathological conditions (54). In 2015, María Yáñez-Mó et al. published an article in the *Journal of Extracellular Vesicles*. This research revealed the critical role of extracellular vesicles in intercellular communication among various cell types. From an immunological perspective, it emphasized the potential of EVs in regulating immune responses and cell-to-cell interactions under physiological and tumor conditions, suggesting their significant clinical potential (56). However, new tools are needed to fully understand their biological composition, function, and uptake mechanisms.

The aforementioned articles elucidate that extracellular vesicles serve as vital carriers of intercellular communication, playing a significant role in the exchange of immune-related information among cells and indicating directions for exploration in this field.

The above-mentioned articles illustrate that extracellular vesicles, as an important carrier of intercellular communication, play a significant role in the exchange of immune-related information between cells, and indicate the direction for exploration in this field.

In 2012, Héctor Peinado et al. published a paper in *Nature Medicine*. This study showed that exosomes promote angiogenesis and increase the permeability of pre-metastatic niches by activating receptor tyrosine kinase MET, thereby influencing the growth and metastasis of melanoma. This reveals how tumors regulate the immune microenvironment through the secretion of extracellular vesicles to promote their growth and spread (55).

In 2014, Suyan Bian and colleagues published in the *Journal of Molecular Medicine* a paper. This study focused on the impact of mesenchymal stem cell (MSC)-derived extracellular vesicles (EVs) on cardiac repair processes. The results showed that these EVs promote cardiac tissue regeneration and prevent ischemic injury by reducing inflammation or activating immune cells related to repair. They also found that EVs from MSCs have therapeutic potential similar to their source cells, promoting endothelial cell proliferation, migration, and angiogenesis (57).

These two articles show the intersection among extracellular vesicles, angiogenesis, and the tumor microenvironment, highlighting the potential value of extracellular vesicles for cancer therapy.

In 2008, Johan Skog and others published in *Nature Cell Biology* a paper. This study clarified that microvesicles from glioblastoma can influence the tumor microenvironment through their carried mRNA, miRNA, and proteins, promoting tumor growth and angiogenesis by interacting with normal host cells. From an immunological perspective, this indicates that tumor cells may use microvesicles to alter the immune environment to escape immune surveillance or suppress immune responses, thus promoting tumor growth. Furthermore, the presence of microvesicles from the tumor immune microenvironment in the serum suggests the potential for blood tests to provide diagnostic and therapeutic information. This study highlights the diagnostic and therapeutic potential of microvesicles in the tumor microenvironment, pointing to a clear direction for the clinical translation of exosomes and microvesicles (53).

These six articles form the knowledge base of this field and point to directions for future research. Next, to identify new hotspots and research trends, we analyzed the top 15 burst-cited references and found two papers that remain highly cited to this day.

The first article, co-authored by Raghu Kalluri et al, found that exosomes are key mediators of intercellular communication found in all biological fluids (68). They can influence cellular behavior and function by transporting DNA, RNA, proteins, and other molecules. From an immunological perspective, exosomes impact both innate and adaptive immunity (45, 69, 70). Exosomes can transfer antigens, MHC molecules, co-stimulatory signals, immune-suppressive molecules (such as PD-L1), and other immune-activating molecules, directly or indirectly affecting the activation status of T cells and other immune cells (71, 72). For instance, exosomes from antigen-presenting cells can directly present antigens to specific T cells to induce their activation. Additionally, by transporting specific miRNAs and non-coding RNAs, exosomes can regulate gene expression within recipient cells, thereby affecting immune cell functions (73, 74). Exosomes are associated with various disease states, including viral infections, cancer, and autoimmune diseases (3, 75, 76). For example, in cancer, exosomes secreted by tumor cells can suppress immune responses and promote tumor growth and metastasis (5). Given their role in diseases and their widespread presence, exosomes are considered potential biomarkers for disease diagnosis and prognosis. Their ability to carry various small molecules, target specific cells, and freely cross the blood-brain barrier also makes exosomes viable tools for targeted drug delivery and as diagnostic tools (77–79). For instance, analyzing exosomes in the blood can detect tumor-associated mutations, proteins, and miRNAs, serving as indicators for diagnosing or monitoring disease progression and treatment response (80–82).

The second article, titled “Exosomes,” emphasized the roles of exosomes in immunosuppression, activation, and intercellular communication (3). For example, exosomes participate in regulating T cell functions and promoting tumor immune evasion through surface molecules like CD39 and CD73 (83–85). Additionally, dendritic cell-derived exosomes can directly kill tumor cells and activate natural killer cells via TNF superfamily ligands (11, 17, 86). These findings demonstrate the potential of exosomes in anti-tumor therapy. Beyond their anti-tumor roles, exosomes also have complex functions in regulating immune responses and disease processes. For example, plasma exosomes with MHC II specificity can suppress inflammation in an antigen-specific and Fas ligand/Fas-dependent manner (87–89). Furthermore, they can directly influence tissue repair by carrying TGF-β1 and HIV-1-related TAR RNA (90–93).

The results of these two articles indicate that the field has progressed from simply focusing on the relationship between exosomes and the tumor microenvironment to exploring specific immune pathways. Combining the analysis of high-centrality keywords from 2021 to 2023, we predict that future research may increasingly focus on using exosomes for targeted drug delivery, employing exosomes as disease biomarkers, and predicting disease prognosis. This could mark a shift from basic research to therapeutic strategies.

### 4.4 Limitation

This study has some limitations. First, all the papers were sourced from the Web of Science Core Collection, potentially overlooking articles not indexed by this database (34). Second, all information was extracted using Citespace 5.8 and VOSviewer 1.6.18, which may cause slight discrepancies compared to studies using other bibliometric software (94). However, our findings are highly consistent with traditional reviews, while also offering more objective, visualized specific information, knowledge networks, and trend hotspots (95).

## 5 Conclusion

Our study conducted a bibliometric analysis of extensive objective data within the field of extracellular vesicle-angiogenesis, using visualization techniques to construct a knowledge map and illustrate the developmental trends in this domain. We explored the significance of extracellular vesicles in regulating the tumor microenvironment and immune system, uncovering their crucial role in tumor evasion through intercellular communication between tumor and immune cells.

Despite unresolved issues in the field, including the precise mechanisms by which extracellular vesicles influence immune responses and the full therapeutic potential of extracellular vesicles, their unique ability to encapsulate and transport nucleic acids, proteins, and lipids during intercellular communication suggests significant clinical potential as disease biomarkers and novel therapeutic approaches. Additionally, emerging analytical techniques like liquid biopsy have enhanced our understanding of the dynamics of extracellular vesicles release and uptake, paving the way for real-time monitoring of disease and therapeutic responses.

## Data Availability

The original contributions presented in the study are included in the article/**Supplementary Material**. Further inquiries can be directed to the corresponding author.
